# Stress, Anxiety, and Depression During Pregnancy: A Survey Among Antenatal Women Attending Primary Health Centers

**DOI:** 10.3390/healthcare12222227

**Published:** 2024-11-07

**Authors:** Sahbanathul Missiriya Jalal, Saad Hamoud Alsebeiy, Nuriya Mousa Jafar Alshealah

**Affiliations:** 1 Department of Nursing, College of Applied Medical Sciences, King Faisal University, Al-Ahsa 31982, Saudi Arabia; 2 Primary Health Center, Health Cluster, Al-Ahsa 31982, Saudi Arabia; 3 Saud Al-Babtain Cardiac Center, Dammam 31463, Saudi Arabia

**Keywords:** stress, anxiety, depression, pregnancy, antenatal women, mental health

## Abstract

**Background/Objectives**: Maternal mental health problems such as stress, anxiety, and depression (SAD) in antenatal women are major public health challenges. This study aimed to determine the levels of SAD in antenatal women and associate the selected variables with them. **Methods:** A cross-sectional study was conducted in selected primary health centers (PHC) in Al-Ahsa, Saudi Arabia. The pregnant women were selected using systematic randomization, and their SAD levels were assessed using the perceived stress scale (PSS), the state anxiety scale (SAS), and the patient health questionnaire (PHQ-9), respectively. Linear regression was used to associate factors related to stress, anxiety, and depression. **Results:** Out of 346 antenatal women, 4% had a high level of stress and 27.2% had a moderate level of stress. Regarding the level of anxiety, 2.6% of them had high anxiety, and 32.9% had moderate anxiety. Around 32 (9.2%) women had moderate depression, and 4 (1.2%) had severe depression. The mean score of SAD was 11.99, 28.88, and 4.73, respectively. A linear regression proved that there was an association between stress and age, occupation, gestational age (GA), gravida, para, abortions, and social support (SS) (*p* < 0.05). Anxiety was associated with age, GA, gravida, para, abortions, past obstetrical complications, and SS (*p* < 0.05). Depression was related to age, education, occupation, para, abortions, past obstetrical complications, and SS (*p* < 0.05). **Conclusions:** Due to the prevalence of SAD in pregnancy, the screening of these conditions and awareness creation about the associated factors can help to identify potential risks earlier and prevent maternal and fetal complications.

## 1. Introduction

The antenatal period is more important to women of reproductive age, which is the starting point of conception and ends at childbirth. Many physiological changes occur within this period, including hormonal status [1]. These changes in hormones within the body will most likely lead to an influx of positive and negative emotions. It is normal and common during pregnancy to experience many emotions [2]. The World Health Organization (WHO) defines maternal mental health as a state of well-being in which a mother realizes her own abilities, can cope with the normal stresses of life, can work productively, and is able to contribute to her community [3]. Maternal mental health issues are widely recognized as a significant public health issue on a global scale. There is an alarming increase worldwide in the prevalence rate of maternal mental health disorders during the antenatal period. According to the WHO report on this finding, approximately 10% of antenatal women and 13% of women who have given birth have previously developed a mental health disorder [4]. Stress, anxiety, and depression (SAD) are the most widely recognized mental health issues during pregnancy. These affect a significant number of pregnant women and result in maternal and childbirth complications. Mental health issues can also be associated with many factors such as family problems, broken homes, issues between the woman and her partner, work–life imbalance, and any other comorbidity conditions [5]. Various research results have shown that the children born to mothers with mental health problems such as SAD were low-birth-weight (LBW) babies [6].

Medical understanding of mental stress involves observing how subjects react to a stimulus and what changes occur because of such a reaction [7]. Mental stressors can affect the well-being of the mother throughout pregnancy. Stress experienced by the mother during pregnancy may interfere not only with the normal progression of the pregnancy, but also the development and behavior of the fetus. This interference can range from mild to severe, depending upon how adverse the effects are [8]. Pre-eclampsia, preterm labor, LBW, and neonatal morbidity are pregnancy outcomes that are directly linked to maternal stress [9]. Furthermore, studies have shown that maternal stress can impact fetal neurodevelopment among children and potentially hinder the formation of socioemotional skills, laying the foundation for their future well-being [10].

Pregnancy anxiety is described as the high levels of concern or fear that a woman may have while she is pregnant. It differs from general anxiety as it is centered on worries related to pregnancy, including the baby’s well-being, childbirth, changes in the body, and parenting a newborn. This kind of anxiety can have a detrimental effect on both the mother and the growing fetus [11]. Unforeseen pregnancies can result in heightened levels of tension and feelings of unpredictability. Financial worries, lack of partner support, or familial issues can worsen anxiety [12]. Anxiety is a prevalent issue in first-time pregnant women, and research has shown that approximately 37–42% of these women have notable anxiety, especially anxiety related to childbirth [13]. Symptoms of anxiety in pregnancy include the following: constant concern about the baby’s health; fear of childbirth or labor complications; worries about lifestyle changes, work–life balance, and parenting; disturbed sleeping; heightened irritability or restlessness; and physical signs such as palpitations, shortness of breath, or tension. Elevated maternal anxiety can lead to adverse childbirth outcomes, including LBW, preterm birth, and developmental delays [14].

Antenatal depression is a term used to describe a period of depression that occurs while a woman is pregnant. It is a significant mental health disorder and has an impact on emotional wellness, physical well-being, and the overall pregnancy journey. During pregnancy, depression can affect both the mother and the growing fetus and may even persist after the baby is born [15]. Persistent sadness, anxiety about the baby or pregnancy, lack of energy, sleep disturbance, loss of interest in activities, feelings of guilt or worthlessness, changes in appetite or weight, difficulty in making decisions, and, in extreme cases, suicidal thoughts or self-harm tendencies are all symptoms of antenatal depression [16]. Changes in hormone levels during pregnancy can impact brain chemistry and mood, leading to the onset of depression. Depression during pregnancy is linked to early birth, LBW, issues with connecting and bonding with the infant, and postpartum depression [17].

Different research studies in Saudi Arabia have found varying rates of maternal stress, revealing high levels of psychological distress in pregnant women. One such study evidenced that numerous prenatal women go through different degrees of stress while pregnant, leading to effects on both their physical and mental well-being. Approximately 41.5% of the women studied were categorized as having a high likelihood of experiencing anxiety, with the most prevalent symptoms being overwhelming concern and dread. Around 37.5% of the participants showed signs of depression, while approximately 25.5% experienced moderate to severe depression and anxiety [18]. Furthermore, a study carried out in Jeddah revealed that around a quarter of pregnant women experienced moderate to severe stress levels while pregnant, underscoring the importance of providing thorough mental health assistance to soon-to-be mothers. Factors such as family support, past childbirth experiences, and medical history can affect the frequency of stress and anxiety in pregnant women [19]. Antenatal women must gain awareness about the importance of mental health and well-being and the impact of poor mental health on the outcome of the mother and the baby. The husband also needs to be educated on these matters, as his parental role is crucial too [20]. Management of mental health issues can include the following: administration of medications such as anxiolytic agents and antidepressants; consultation with therapists; relaxation; social support (SS) from the husband, close friends, and family; receiving couple counseling; and techniques for relieving stress [21]. Anxiety during pregnancy is a significant public health concern that needs to be addressed for improved outcomes for both mother and baby. Incorporating mental health screenings into regular antenatal care can identify women in need and thus offer timely support. Understanding how common and significant pregnancy depression is crucial for addressing maternal mental health and enhancing pregnancy results [22].

Previous research reports have shown that mental health issues are common, and these issues have a negative impact during pregnancy. The novelty of this present study is that it is region-specific and contributes unique insights into psychological well-being by using appropriate SAD scales among antenatal women for a comprehensive assessment to find different stressors and influences compared to other studies. Hence, the purpose of this study is to assess the levels of SAD among antenatal women and to determine the associations between these mental health conditions and the selected variables. This study results will bring attention to the importance of early screening and awareness to reduce the risk of complications in maternal and fetal health through examining the prevalence of these mental conditions and their associated factors.

## 2. Materials and Methods

### 2.1. Study Design

Using a descriptive method, a cross-sectional study design was conducted to assess the level of SAD in pregnancy period among antenatal women, and its associations with the selected variables. In accordance with STROBE (Strengthening the Reporting of Observational Studies in Epidemiology) criteria, this survey was carried out between September 2023 and March 2024 after obtaining ethical clearance.

### 2.2. Study Area and Setting

This study was conducted in Al-Ahsa, a region located in the eastern province of Saudi Arabia, known for its blend of urban and rural populations. There are 72 primary health centers (PHC) functioning in this area. The setting included selected PHC across the region, chosen for their antenatal visit rates and accessibility. These centers provide comprehensive antenatal care services, which are ideal for assessing the mental health of pregnant women. Approximately 1,300,000 people live in this area, with nearly half being female [23].

### 2.3. Sample Size and Sampling

Sample size was calculated using the website openepi.com, with a margin of error of 5%, confidence level of 95%, and population size of 55,900. Due to the absence of precise prevalence data, assuming a 50% prevalence is common for calculating sample sizes in public health research, as it maximizes sample size requirements and ensures adequate statistical power. Our calculations showed that the minimum sample size should be 376 [24]. By using cluster technique, five PHCs were randomly selected, with one PHC chosen from each of the five zones (northern, southern, western, eastern, and central) which led to a higher variance in estimates and reduced precision. The sampling error for each cluster, with a sample size of 70 and a cluster population size of 11,180, was approximately 11.68%. From each PHC, 70 antenatal women (every 4th woman) visiting the PHC for regular antenatal checkup were selected by systematic random sampling. Considering the inclusion criteria, a total of 346 antenatal women were included in the data collection.

### 2.4. Inclusion and Exclusion Criteria

Antenatal women who were aged from 19 years onwards and who were in the active reproductive stage, citizens of Saudi residing in Al-Ahsa, who were attending the selected PHC, who showed interest in participation of research, and who agreed for data collection, were included in this study. Pregnant women at any gestational age (GA), whether they were primigravida or multigravida, were included in the data collection. The exclusion criteria for this study included pregnant women with severe chronic diseases, obstetrical complications, or a documented history of psychiatric disorders, as these conditions could confound assessments of pregnancy-related SAD.

### 2.5. Data Collection Tool, Procedure, and Quality Control

A well-structured, validated questionnaire was used as a self-administered tool to gather data from antenatal women to assess SAD. The questionnaire was developed that included four components, with the first part focused on the socio-demographic variables of the participants. These socio-demographic data included age, educational qualifications of the pregnant women, educational qualifications of their husbands, occupation, GA, gravida, para, number of previous abortions, history of previous obstetrical complications, and SS. The second component of the tool focused on stress, which was assessed using the perceived stress scale (PSS-10). The third component addressed the anxiety levels of pregnant women, which was assessed using the state anxiety scale (SAS). The fourth component covered depression in pregnant women, and the patient health questionnaire (PHQ-9) was used to assess this. The data collection tool was originally developed in English and subsequently translated into Arabic. To assess the tool’s validity, clarity, and feasibility, a structured questionnaire (with both English and Arabic versions) was reviewed by a panel of experts, including three obstetricians, two maternal nurse specialists, and three family physicians experienced in antenatal care at PHC. The tool modifications were made based on their feedback. A preliminary study was conducted with 15 menopausal women to assess the reliability and usability of the tool. Also, reliability was measured using Cronbach’s alpha, resulting in a coefficient of 0.856, which indicated good internal consistency. Participants required approximately 25–30 min to complete the survey. Prior to the data collection, the study’s objectives were clearly explained to all participants. Data from completed questionnaires were double-checked during entry to minimize errors and maintain data accuracy.

#### 2.5.1. Perceived Stress Scale

The PSS-10 is designed to assess individuals’ perceptions of stress in their lives [25]. This brief evaluation consists of 10 questions measuring stress experienced over the past 4 weeks. Each item is rated on a five-point Likert scale ranging from 0 (Never) to 4 (Very Often). Higher scores indicate a higher perceived stress level. The scoring categories are as follows: low stress (0–13), moderate stress (14–26), and high stress (27–40).

#### 2.5.2. State Anxiety Scale

The SAS questionnaire consists of 20 items [26] where respondents indicate how they feel “right now, at this moment”. Each item is rated on a four-point Likert scale, typically with the following range: 1 = Not at all; 2 = Somewhat; 3 = Moderately; and 4 = Very much so. The total possible score ranges from low-state anxiety (20) to high-state anxiety (80). The interpretation of scores are as follows: 20–30, low-state anxiety; 31–40, moderate-state anxiety; 41–50, high-state anxiety; and 51–80, very-high-state anxiety.

#### 2.5.3. Patient Health Questionnaire

The patient health questionnaire (PHQ-9) is a screening tool designed to assess the presence of depressive symptoms [27]. It was utilized to evaluate the severity of depressive symptoms in pregnant women. The scoring system ranges from 0 to 4, indicating minimal depression; from 5 to 9 for mild depression; from 10 to 14 for moderate depression; from 15 to 19 for moderately severe depression; and from 20 to 27 for severe depression. A score of 10 or higher is regarded as the threshold for diagnosing depression. This tool is both reliable and valid for identifying symptoms of prenatal depression.

### 2.6. Ethical Considerations

This research was carried out following ethical principles in line with the Declaration of Helsinki. Approval for this study was ethically granted by the Research Ethics Committee of the Deanship of Scientific Research at King Faisal University in Al-Ahsa, Saudi Arabia, in June 2023 (Reference No. 964). Before data collection commenced, the information was given to them about the privacy of their answers, that there were no risks involved, that their participation was anonymous, and that their involvement was voluntary. All antenatal women involved in this study were required to provide written consent. Once the inclusion criteria were evaluated, all participants were clearly briefed on the study’s goals and research intentions during data collection. They were allowed to exit the study at any point based on their preferences. All participants were guaranteed that their information would be kept private.

### 2.7. Statistical Analysis

Statistical analysis was performed using SPSS software (version 21.0; IBM, Armonk, NY, USA) to evaluate the data collected in this study. Descriptive statistics included frequencies and percentages to examine individual variables and their mental health issues, while the mean score was calculated for SAD levels. In inferential statistics, linear regression tests were used to analyze the association between SAD levels in antenatal women and the selected demographic variables, with a 95% confidence interval. The Kruskal–Wallis H test was applied to assess significant differences in SAD levels. Pearson’s correlation test was utilized to identify the relationship between depression, stress, and anxiety. A *p*-value of 0.05 or less was considered statistically significant.

## 3. Results

### 3.1. Demographic Variables of Antenatal Women

A total of 346 antenatal women were included in the analysis (Table 1), of which 187 (54%) were in the age range of 19–29 years. The mean age of the women was 30.42 (SD ± 5.42) years. Approximately 144 (41.6%) of the antenatal women studied up to high school level. Regarding the educational level of their husbands, 123 (35.5%) graduated from universities. Most of the antenatal women (184, 53.2%) were either housewives or studying. Regarding the gestational age, 134 (38.7%) were in their second trimester. The majority (163; 47.1%) were either primigravida or second gravida. Around 16 (4.6%) of the women were in three or more parities. The mean score of GA was 5.03 (SD ± 2.25) months, gravida was 2.59 ± 1.59, and para was 1.16 ± 1.22. Most of the antenatal women (253; 73.1%) did not have an abortion, while 21 (6.11%) had more than one abortion. Among the women, 107 (30.9%) had a previous history of obstetrical complications. Regarding social support, 76 (22%) had no support from anyone, 104 (30.1%) had support from partners, and 114 (32.9%) received support from their parents.

### 3.2. Prevalence of Mental Health Issues of Antenatal Women

The frequency distribution of mental health issues (SAD) among women is shown in Table 2. Out of 346 antenatal women, 14 (4%) had a high level of stress and 94 (27.2%) had a moderate level of stress. Regarding the level of anxiety, 9 (2.6%) had high anxiety, whereas 114 (32.9%) had moderate-level anxiety. Regarding depression, 100 (28.9%), 32 (9.2%), and 4 (1.2%) antenatal women had mild, moderate, or severe depression, respectively. The mean score for stress levels was 11.99, for anxiety was 28.88, and for depression was 4.73 (Table 3). The Kruskal–Wallis H test indicated that there were significant differences in mental health issues (SAD) among the three groups, χ^2^(2) = 670.95, *p* < 0.001, with a mean rank score of 478.54 for stress, 832.32 for anxiety, and 247.63 for depression.

### 3.3. Association of Mental Health Issues of Antenatal Women with Selected Variables

A multiple regression model explored the relationship between various predictor variables and outcomes, potentially measuring SAD during pregnancy. A linear regression analysis was conducted to assess the relationship between stress levels and socio-demographic variables among 346 antenatal women (Table 4). The model explained a significant portion of the variance in stress levels, as evidenced by the significant predictor variables. Age was positively associated with stress levels (B = 1.692, *p* = 0.012), indicating that as maternal age increased, stress levels also tended to rise. The education level of the participants had no significant effect on stress (B = −0.849, *p* = 0.092), suggesting that educational attainment did not strongly predict stress in this study. Education level of the husband was also not a significant predictor (B = 0.467, *p* = 0.363). Occupation was significantly associated with stress (B = 2.073, *p* < 0.001), where being employed or engaged in an occupation was linked to lower stress levels. GA showed (B = 0.492, *p* = 0.070) a weak relationship between advancing pregnancy and stress levels. Gravida (number of pregnancies) was a significant predictor (B = 2.302, *p* < 0.001), with stress increasing as the number of pregnancies increased. Para (number of births) showed a strong positive association with stress levels (B = 2.793, *p* < 0.001), meaning that women with more previous births experienced higher stress levels. Abortion history was also a significant predictor (B = 6.392, *p* < 0.001), with women with a history of abortion reporting higher stress levels. Past obstetrical complications were not significantly associated with stress levels (B = 0.148, *p* = 0.833), suggesting that those with previous complications might manage stress differently. SS was a significant protective factor against stress (B = −1.938, *p* < 0.001), with higher SS leading to lower stress levels.

### 3.4. The Association Between the Stress Level of Antenatal Women

In Table 5, the relationship between anxiety levels and socio-demographic variables among 346 antenatal women were shown. Age had a strong positive association with anxiety, which indicated (B = 4.677, *p* < 0.001) that higher age was associated with higher anxiety scores. The mother’s education level showed a weak and statistically insignificant association with anxiety (*p* = 0.638). Similarly, the education level of the husband was not significantly related to anxiety (*p* = 0.761). The occupation was non-significant with anxiety (*p* = 0.4). GA was a significant positive predictor (0.001). The number of pregnancies (gravida) showed a positive and significant effect (*p* = 0.029), suggesting that women with more pregnancies were likely to experience higher levels of anxiety. The parity of women was significant (*p* < 0.019), indicating that a higher number of live births was associated with higher levels of anxiety. Experience of abortion was significantly (*p* = 0.0234) associated with higher anxiety. Past obstetrical complications were associated significantly with levels of anxiety (*p* < 0.005). SS also has a significant negative effect (*p* < 0.001), indicating that higher levels of SS reduced the level of anxiety.

Table 6 presents the results of a linear regression analysis which examined the association between depression scores and various socio-demographic variables in a sample of 346 antenatal women. Age had a significant positive effect on the PHQ score, meaning that as age increases, the PHQ score also increased (1.355, *p* < 0.001). Higher education was positively associated with the PHQ score, suggesting that individuals with more education have higher PHQ scores (1.176, *p* < 0.001). Husband’s education was negatively associated with PHQ scores, although this was weakly significant (−0.590, *p* = 0.048). Being employed (likely) decreased PHQ scores (−0.974, *p* = 0.002). There was no significant relation between depression with GA (−0.075, *p* = 0.634) and gravida (0.214, *p* = 0.555). However, the number of live births (2.168, *p* < 0.001), history of abortion (2.442, *p* < 0.001), and previous obstetrical complications (1.179, *p* = 0.004) significantly related to depression. SS had a significant negative effect on depression scores (−0.794, *p* < 0.001), meaning that adequate support from their partner, family members, and friends were associated with lower depression.

Pearson’s correlation test indicated that (Table 7) there was a significant large positive relationship between depression and stress, (*r* (344) = 0.782, *p* < 0.01). There was a significant large positive relationship between depression and anxiety (*r* (344) = 0.786, *p* < 0.01).

## 4. Discussion

This study’s results offer an understanding of the mental health challenges experienced by pregnant women, focusing on SAD and the factors linked to these issues. A survey conducted to analyze the occurrence and factors of depression and anxiety in pregnant women during the final trimester revealed the significance of tracking mental well-being in pregnancy and of recognizing key risk elements in order to offer suitable support, as well as the necessity for interventions promoting mental wellness in expectant mothers [28]. Our study included antenatal women aged from 19 years onwards, who had a mean age of 30.42 years. Similarly, another study conducted in China included participants who were aged between 16 and 44 years and who had an average gestational age of 10.7 weeks. Almost half of these women still worked after pregnancy and had a college degree or above [29].

In this present study, the results showed that a significant number of antenatal women experience mental health issues. About 4% of the women had high levels of stress, and 27.2% had moderate stress. Anxiety was also prevalent: 32.9% experienced moderate anxiety and 2.6% experienced high anxiety. In addition, depression affected many of the participants: 28.9% had mild depression and 9.2% experienced moderate depression. A separate study was conducted to investigate the pathways to probable and severe depression in pregnant women in Spain, aiming to identify the associated and predictive factors. The findings regarding probable and severe depression pathways were consistent with our research on pregnancy. Throughout all three trimesters, psychological factors, including perceived stress, were associated with both forms of depression [30].

The current study reveals several socio-demographic factors associated with stress levels among antenatal women. Maternal age was positively correlated with stress, suggesting that older women may experience more stress during pregnancy. Some studies support this observation. Research has found that women of advanced maternal age are more likely to report higher levels of stress due to both medical and non-medical factors [31]. Similarly, a study emphasized that older pregnant women often face greater expectations and pressures from both them and their environment, which may compound their feelings of stress. This may be because older women often have more life responsibilities, such as established careers or other children, and therefore experience higher psychological demands [32].

Women with more pregnancies (gravida) and more live births (para) also experienced higher stress levels in this study. This could be attributed to increased responsibilities and concerns regarding the health of both the mother and the baby. The burden of childcare, combined with the physiological demands of pregnancy, may heighten anxiety and stress levels. According to another study, multiparous women are more likely to experience cumulative stress due to increased family obligations, which can lead to psychological strain and diminished emotional well-being during subsequent pregnancies [33]. Furthermore, in our present study, women with a history of abortion had significantly higher stress levels, likely due to concerns about the outcomes of their current pregnancy. This psychological burden might be exacerbated by societal stigmas or personal beliefs about abortion, leading to feelings of shame or isolation, which, in turn, contribute to elevated stress levels, as indicated in another study [34]. On the other hand, SS was found to be a protective factor, as women with better support systems reported lower levels of stress in that same study. This observation is supported by another study, in which the mechanisms by which SS reduces stress are found to be multifaceted. Emotional support provides reassurance and a sense of belonging, while instrumental support, such as assistance with daily tasks, alleviates the physical and mental demands of pregnancy. In addition, supportive relationships may foster positive coping strategies and enhance an individual’s perceived ability to manage stressors [35].

Age was also positively associated with anxiety levels. Women in more advanced stages of pregnancy and those with more pregnancies and live births showed higher levels of anxiety in this study. This aligns with previous findings suggesting that, as pregnancy progresses, concerns related to childbirth, health of the fetus, and personal well-being intensify, leading to heightened anxiety levels. Several studies have highlighted that increased gravidity and parity are often associated with higher psychological stress and anxiety, likely due to past experiences with childbirth or complications in previous pregnancies [36,37,38]. Additionally, women with a history of abortion or past obstetrical complications were more likely to experience anxiety in this current study. Previous research has demonstrated that women with a history of obstetric complications are more likely to report heightened anxiety levels due to fears related to the recurrence of these complications and the health of their current pregnancy [39,40,41]. SS, once again, played a crucial role in reducing anxiety in the same study, indicating the importance of emotional and practical assistance from family and partners in alleviating mental health burdens. Previous studies have also demonstrated that women who were supported by their families were more likely to develop positive coping strategies, leading to better psychological outcomes during pregnancy [42].

In this current research, age and education level were significantly associated with higher depression scores, suggesting that older women and those with higher educational levels may face more psychological pressures. This relationship is consistent with the existing literature, which suggests that both age and education can impact mental health during pregnancy [43,44]. As with stress and anxiety, the number of live births, history of abortion, and past obstetrical complications were significant predictors of depression. This is supported by few other research studies; women who experience complications may fear a recurrence, leading to increased emotional distress and depressive symptoms [45,46]. SS once again emerged as a critical factor in reducing depression, reinforcing the need for comprehensive support systems for pregnant women in that study. This is consistent with prior research, which indicates that emotional and practical support from partners, family, and friends can buffer against the psychological stressors that pregnant women often face [22].

Overall, these findings highlight the multidimensional nature of mental health among antenatal women. Socio-demographic factors, obstetric history, and SS all play significant roles in influencing SAD during pregnancy. Older pregnant women, especially those beyond typical childbearing years, may experience heightened SAD due to increased physical risks and societal expectations regarding maternal age. For women with repeated pregnancies or miscarriages, prior complications can cause anticipatory anxiety about adverse outcomes, which may escalate stress levels. In pregnant women, emotional and practical support from family, friends, or community organizations may alleviate anxieties and depressive symptoms by providing reassurance, sharing responsibilities, and fostering a sense of stability. The results underlined the importance of integrating mental health services into antenatal care, ensuring that women in need to receive adequate support throughout their pregnancies. Healthcare providers should focus on identifying women at risk of mental health issues, especially those with a history of multiple pregnancies, abortions, or limited SS, and provide appropriate interventions.

The strength of this study is its employment of a cross-sectional design, which assessed the mental health status of antenatal women during a defined period. This provided valuable insights into the prevalence of SAD among antenatal women. This study had sufficient statistical power to detect significant associations between variables, enhancing the generalizability of the findings within context. This study also utilized validated questionnaires (the PSS-10, SAS, and PHQ-9) to measure SAD levels. However, some limitations of this study were found. The causality between the variables was not studied, as this is a cross-sectional study. The exclusion of pregnant women with severe chronic diseases may limit the understanding of the mental health status of antenatal women. Limiting the study setting to the PHC in Al-Ahsa, Saudi Arabia, may influence the generalizability of the findings. However, these findings can serve as a basis for further research in other areas, which could help determine if similar associations exist elsewhere.

## 5. Conclusions

In summary, the prevalence of SAD among antenatal women is concerning, and various socio-demographic and obstetric factors contribute to these mental health issues. Age, occupation, gravida, para, abortion history, past obstetrical complications, and SS were all found to be significant variables affecting stress levels during pregnancy. Receiving support from a partner, family, or friends emerged as a strong protective factor, indicating that higher levels of SS reduce the outcome of SAD. The results of this study suggested that these factors should be considered in mental health interventions for pregnant women. These interventions, including counseling and SS, are important for reducing the mental health burdens and improving the overall well-being of antenatal women.

## Figures and Tables

**Table 1:** Frequency distribution of variables in antenatal women (*n* = 346).

Demographic Variables		Number	Percent
Age	19–29	187	54.0
	30–39	136	39.3
	≥40	23	6.6
Education level	Primary school	15	4.3
	Middle school	85	24.6
	High school	144	41.6
	University	102	29.5
Education level of Husband	Primary school	12	3.5
	Middle school	76	22.0
	High school	135	39.0
	University	123	35.5
Occupation	Unemployed	184	53.2
	Employed	162	46.8
Gestational age	I trimester	123	35.5
	II trimester	134	38.7
	III trimester	89	25.7
Gravida	1–2	163	47.1
	3–4	137	39.6
	>4	46	13.3
Para	0–1	254	73.4
	2–3	76	22.0
	>3	16	4.6
Abortion	0	253	73.1
	1	72	20.8
	>1	21	6.1
Past obstetrical complications	No	239	69.1
	Yes	107	30.9
Social support	None	76	22.0
	Partner	104	30.1
	Family	114	32.9
	Friends	52	15.0

**Table 2:** Frequency distribution of mental health issues in antenatal women (*n* = 346).

Mental Health Issues	Category	Score	Number	Percent
Stress	Low stress	0–13	238	68.8
	Moderate stress	14–26	94	27.2
	High stress	27–40	14	4.0
	Low anxiety	20–30	204	59.0
Anxiety	Moderate anxiety	31–40	114	32.9
	High anxiety	41–50	19	5.5
	Very high anxiety	51–80	9	2.6
Depression	Minimal depression	0–4	198	57.2
	Mild depression	5–9	100	28.9
	Moderate depression	10–14	32	9.2
	Moderate severe depression	15–19	12	3.5
	Severe depression	20–27	4	1.2

**Table 3:** Kruskal–Wallis H test on mental health issues of antenatal women (*n* = 346).

Mental Health Issues	Mean Score	Median	Skewness	Excess Kurtosis	Kruskal–Wallis H test
Stress	11.99	9	0.8769	0.1092	χ^2^(2) = 670.95 *p* < 0.001 *
Anxiety	28.88	24	1.2323	1.9359	
Depression	4.73	3	1.9349	4.0168	

* Significant.

**Table 4:** Linear regression of stress level and socio-demographic variables (*n* = 346).

Model	B	Std. Error	Beta	*t*	Significance
(Constant)	−5.001	1.353		−3.698	<0.001 *
Age	1.692	0.673	0.122	2.516	0.012 *
Education level	−0.849	0.503	−0.084	−1.689	0.092 NS
Education level of Husband	0.467	0.513	0.046	0.910	0.363 NS
Occupation	2.073	0.548	0.121	3.779	<0.001 *
Gestational age	0.492	0.271	0.045	1.818	0.070 NS
Gravida	2.302	0.623	0.188	3.694	<0.001 *
Para	2.793	0.721	0.181	3.873	<0.001 *
Abortion	6.392	0.556	0.436	11.504	<0.001 *
Past Obstetrical complications	0.148	0.700	0.008	0.211	0.833 NS
Social support	−1.938	0.231	−0.224	−8.393	<0.001 *

Dependent variable: level of stress; B—unstandardized predictor; * significant; *p* < 0.05; NS—non-significant.

**Table 5:** Linear regression of anxiety level and socio-demographic variables (*n* = 346).

Model	B	Std. Error	Beta	*t*	Significance
(Constant)	9.410	1.530		6.151	<0.001 *
Age	4.677	0.761	0.341	6.147	<0.001 *
Education level	0.268	0.569	0.027	0.471	0.638 NS
Education level of Husband	−0.176	0.580	−0.018	−0.304	0.761 NS
Occupation	0.523	0.620	0.031	0.843	0.400 *
Gestational age	1.016	0.306	0.093	3.317	0.001 *
Gravida	1.541	0.705	0.127	2.187	0.029 *
Para	1.915	0.816	0.125	2.347	0.019 *
Abortion	3.387	0.629	0.234	5.389	<0.001 *
Past obstetrical complications	2.247	0.791	0.123	2.839	0.005 *
Social support	−1.300	0.261	−0.152	−4.980	<0.001 *

Dependent variable: level of anxiety; B—unstandardized predictor; * significant; *p* < 0.05; NS—non-significant.

**Table 6:** Linear regression of depression score and socio-demographic variables (*n* = 346).

Model	B	Std. Error	Beta	*t*	Significance
(Constant)	−3.520	0.785		−4.483	<0.001 *
Age	1.355	0.390	0.192	3.469	<0.001 *
Education level	1.176	0.292	0.228	4.029	<0.001 *
Education level of Husband	−0.590	0.298	−0.114	−1.982	0.048 *
Occupation	−0.974	0.318	−0.111	−3.058	0.002 *
Gestational age	−0.075	0.157	−0.013	−0.476	0.634 NS
Gravida	0.214	0.362	0.034	0.591	0.555 NS
Para	2.168	0.419	0.276	5.178	<0.001 *
Abortion	2.442	0.323	0.328	7.569	<0.001 *
Past Obstetrical complications	1.179	0.406	0.125	2.903	0.004 *
Social support	−0.794	0.134	−0.180	−5.921	<0.001 *

Dependent variable: level of depression; B—unstandardized predictor; * significant; *p* < 0.05; NS—non-significant.

**Table 7:** Relationship between levels of depression and levels of stress and anxiety among antenatal women (*n* = 346).

Mental Health Issues	Depression		
	Correlation Coefficient	Covariance	Significance (2-Tailed)
Stress	0.7819	29.3062	*p* < 0.01 *
Anxiety	0.7861	29.1134	*p* < 0.01 *

* Significant.

## Data Availability

Data is contained within the article.

## Abbreviations

GA	Gestational age
LBW	Low birth weight
PHC	Primary health center
PHQ	Patient health questionnaire
PSS	Perceived stress scale
SAD	Stress, anxiety, and depression
SAS	State anxiety scale
SD	Standard deviation
SPSS	Statistical Package for Social Sciences
SS	Social support
STROBE	Strengthening the reporting of observational studies in epidemiology
WHO	World Health Organization
